# DEPICTION OF OLDER ADULTS IN AN INTERNATIONAL PACIFIC RING STUDENT COMPETITION ON OLDER ADULT CARE

**DOI:** 10.1093/geroni/igad104.2322

**Published:** 2023-12-21

**Authors:** Jonathan Guillemot, Raudah Mohd Yunus, Carlos Joaquin Rueda Ordoñez, Mellissa Withers, Meiqi Xin, Mary Rozelle, Ksenia Kubasova, Noran Naqiah Hairi

**Affiliations:** Universidad San Francisco de Quito, Quito, Pichincha, Ecuador; Universiti Teknologi MARA, Shah Alom, Selangor, Malaysia; Universidad San Francisco de Quito, Quito, Pichincha, Ecuador; University of Southern California, Los Angeles, California, United States; Hong Kong Polytechnic University, Hong Kong, Hong Kong; University of Southern California, Los Angeles, California, United States; NA, NA, Moskva, Russia; Universiti Malaya, Kuala Lumpur, Kuala Lumpur, Malaysia

## Abstract

The Global Health Program of the Association of Pacific Rim Universities (APRU), a consortium of universities in countries surrounding the Pacific Ocean, organized a competition in 2020 named “2020 Case Competition Challenge “Improving Elderly Care in the Asia-Pacific””. It yielded proposals from 28 teams from nine countries in Asia and the Americas in the form of 10-minute videos. Our study aimed to analyze the depiction of older adults to identify patterns of portrayal of older adults among university students. We coded the videos according to a two-step process, whereby we first filtered the videos to identify extracts related to the portrayal of older adults and subsequently coded the dataset according to a list of 10 codes, which were elaborated iteratively during the first step of coding. We analyzed the coded data and identified patterns, which served as a basis for analytical axes. Across included economies, we identified strong patterns of discriminatory discourse and depiction. Older adults were often assumed to be systematically sick, disconnected from the social and technological realities as well as incapable of being active contributors to society. Teams often proposed solutions medicalizing socialization, whereby social interactions were represented as clinical interventions void of meaning. In this unusual approach to studying portrayal of older adults among university students, we found concerning patterns of bias, therefore calling for immediate action towards tackling discrimination at university levels. Considering the pace of global aging, such intervention would contribute the essential view that demographic aging, as such, is not a problem.

